# Occupational health and safety in mining: Predictive probabilities of Personal Protective Equipment (PPE) use among artisanal goldminers in Ghana

**DOI:** 10.1371/journal.pone.0257772

**Published:** 2021-09-30

**Authors:** Simon Appah Aram, Benjamin M. Saalidong, Augustine Appiah, Idongesit Bassey Utip

**Affiliations:** 1 Research Center for Smart Mine and Intelligent Equipment, Taiyuan University of Technology, Taiyuan, People’s Republic of China; 2 College of Safety and Emergency Management Engineering, Taiyuan University of Technology, Taiyuan, People’s Republic of China; 3 Department of Geosciences, Taiyuan University of Technology, Taiyuan, People’s Republic of China; National Research Centre of Egypt, EGYPT

## Abstract

Artisanal goldminers in Ghana are exposed to various levels and forms of health, safety and environmental threats. Without the required legislation and regulations, artisanal miners are responsible for their own health and safety at work. Consequently, understanding the probabilities of self-protection at work by artisanal goldminers is crucial. A cross-sectional survey of 500 artisanal goldminers was conducted to examine the probabilities of personal protective equipment use among artisanal goldminers in Ghana. The data was subjected to both descriptive and inferential statistics. Initial findings showed that personal protective equipment use among artisanal miners was 77.4%. Overall, higher probabilities of personal protective equipment use was observed among artisanal goldminers who work in good health and safety conditions as compared to artisanal miners who work in poor health and safety conditions. Also, personal protective equipment use was more probable among the highly educated artisanal goldminers, miners who regularly go for medical screening and the most experienced miners. Additionally, personal protective equipment use was more probable among artisanal miners who work in non-production departments and miners who work in the medium scale subsector. Inversely, personal protective equipment use was less probable among female artisanal miners and miners who earn more monthly income ($174 and above). To increase self-care and safety consciousness in artisanal mining, there is the need for a national occupational health and safety legislation in Ghana. Also, interventions and health promotion campaigns for better occupational conditions in artisanal mining should target and revise the health and safety related workplace programs and conditions.

## Introduction

Artisanal goldmining is on the rise globally due to the rising prices for minerals and precious metals [1, 2]. There are about 20 million people who work directly in artisanal mining, and over 80 million depend on it for their livelihood [1]. The goldmining industry in Ghana is one of the most important contributors to the nation’s economy in terms of employment, direct and indirect revenues, exports, and investment [3]. Ghana is the largest gold producer in Africa and the seventh largest in the world [4]. As such, Ghana’s mining sector attracts much attention from large scale, medium scale and small scale mining in both local and international fronts. In Ghana, artisanal gold mining accounts for over 20% of the nation’s gold production and render employment to thousands of people mostly indigenous folks [5].

Despite these massive contributions, artisanal mining is generally associated with hazardous working conditions which affect the health, safety and environment (HSE) of its workers [6]. The sector is regarded as one of the health and safety critical domains coupled with dangerous operations within an environment where workers are exposed to abundant risks and hazards [7]. In Ghana, it is publicly known that artisanal miners operate in unsafe conditions which pose serious threats to everyone engaged in it [8]. The operations of artisanal miners present considerable amount of risks at an escalating rate [9]. The processes involved in this activity pose many occupational HSE hazards. The most common of these are mercury exposure, airborne silica dust and fumes generated from chiseling, drilling, blasting, grinding and crushing from pulverized ore, and noise from mining equipment [1].

The reliance on rudimentary techniques and the limited injection of capital investment in the sector places it at a disadvantage in terms of HSE conditions of its miners as compared to their largescale counterparts [10]. Accidents in artisanal mining occur frequently and are expected to occur [11]. However, most artisanal small scale (ASM) and medium scale (AMM) companies do not engage qualified HSE personnel or officers to manage these occurrences. Concession owners in this case sometimes work as supervisors or managers [12]. This place the miners in a more dangerous HSE situation emanating from the lack of in-depth appreciation of the processes and the risks involved with their operations, and accident causation models and theories [13].

There is lack of adherence to good HSE practices and as a result these miners frequently suffer accidents, which sometimes lead to deaths, permanent disability, or reduced quality of life [14]. There is lack of safety regulations and enforcement, education and training, and functional infrastructure and equipment due to the absence of safety officers in the work place of some of these mines [5]. As a result, these miners are liable for their own health and safety at work. Adhering to standard HSE practices in artisanal mining is largely born out of individual’s knowledge, beliefs and perception. It has been acknowledged that the lack of HSE culture in artisanal mining accounts for the majority of unsafe acts and conditions [15].

Advancing policies that improve HSE conditions and culture in artisanal mining requires a comprehension of the main causes of factors that influence them. How miners conduct themselves, in this case the use of personal protective equipment (PPEs) at work, is decided by several factors which are entrenched in institutional contexts, socio-cultural norms and situations [16]. In the absence of effective risk awareness in artisanal mining, it appears most miners are likely to evaluate risk based on their compositional attributes and working conditions at the workplace. This study sought to examine the factors that contribute to artisanal miners usage of PPEs at work even in the absence of supervisory powers and regulations using Generalized Linear Models (complementary log-log logistic regression model). With this model, the effects of occupational factors on PPE use can be assessed while controlling for relevant compositional and contextual factors.

## Methods

### Source of data and sampling procedure

The study location is the Southwestern part of Ghana. This area has the highest per capita concentration of goldminers in Ghana. Twenty known and accessible artisanal mine sites were visited for the survey. Data collection took place from January 2018 to December 2019. A multistage sampling involving stratified and simple random procedures were adopted to ensure a fair and higher probability of the selection of each sampling unit. Artisanal miners considered in this study were registered small scale and medium scale groups. Due to ethical considerations and the reliance on self-reported indicators, participants who had worked for less than a month and participants less than 18 years were not recruited.

### Data collection methods

The questionnaire, developed from other related studies [see, 16], comprised of closed-ended questions and was structured into occupational characteristics (HSE quality measures) and socio-demographic aspects. Scale reliability (Cronbach’s a) of the instrument was 0.788. Respondents had multiple-choice answers to select as applicable. A pre-test was conducted among 50 participants with similar socioeconomic background to the respondents of the study to test for validity and reliability. This led to minor modifications of the questionnaire to improve understanding. Overall, 500 artisanal miners were recruited based on a 95% confidence interval, 50% estimated population proportion and at a 5% error rate.

### Measures

#### Response variable

The outcome variable in this study was “PPE use” by artisanal goldminers. Goldminers’ job characteristics and environment were assessed to know the protective equipment required to protect themselves at work. Each miner was then observed and also asked about the protective equipment they used while working. If the miner missed any of the required equipment, PPE use was indicated as “No” and if they used the complete set of equipment it was indicated as “Yes”. Complete set of PPEs in this study meant a miner had protective equipment for head, hearing, eye, respiratory, hand, foot and body.

#### Occupational factors

The occupational factors (key predictor) selected for this study were self-rated (i) health conditions, (ii) safety conditions and (iii) environmental conditions of goldminers. These variables were evaluated as very poor (1), poor (2), good (3), very good (4), and excellent (5)”, with a series of questions. Total scores greater than 3 for each variable was considered as “good” and scores less than 3 as “poor”. In this study, “Health conditions” implied the functional status of gold miners and other occupational related health threats. These include work-related injuries and diseases, emotional wellbeing, fatigue, stress, provision of first aid and the rate of change in health status. “Safety conditions" here refers to availability of appropriate PPEs, competent safety officers, safety training, work process, risk assessment and hazard identification, institutionalization of practices such as allowed levels of noise and protection against fall in accordance with standard safety practices. “Environmental conditions” encompasses both physical and social factors. These included but were not limited to appropriate tools for the job, perceptions of their workspace quality and setting, working environment and space availability.

#### Compositional and contextual factors

Compositional factors chosen for this study were age, sex, marital status, education, years of experience, income and routine medical screening. For routine medical screening, goldminers were quizzed on how frequently they go for voluntary medical screening in a year; miners who indicated a minimum of 1 were categorized as “Yes” and those who indicated none in a year were categorized as “No”. The contextual factors were work department and subsector of the goldminer.

The selection of these occupational, compositional and contextual factors were based on theoretical relevance, practical significance and parsimony.

### Data analyses

The data was subjected to univariate, bivariate and multivariate statistical analyses to examine the relationships and proportions between factors that influence PPE use in artisanal goldmining. All statistical analyses were performed using Stata 15 (StataCorp, College Station, Texas) SE software at a statistical significance of 0.05 and at a confidence interval of 95%.

#### Univariate and bivariate analyses

The percentages and distributions of the characteristics of the goldminers was determined with the univariate analysis. Pearson chi-square and Cramer’s V statistics were used to test and describe the relationship between the categorical independent variables and PPE use at work by artisanal goldminers.

#### Multivariate analyses

The effects of occupational factors, compositional and contextual attributes on PPE use among artisanal goldminers were determined using complementary log-log logistic regression model. Logistic regression allows the model to be related to the response variable via a link function and by allowing the magnitude of the variance of each measurement to be a function of its predicted value under the assumption of binary response (Yes/No). In this study, 77.40% of artisanal miners used PPE hence, the complementary log-log link function was operationalized.

In the multivariate analysis, the practical significance of the findings was also estimated by using predictive probabilities. The predictive probabilities calculated were adjusted predictions referred to as margins in Stata 15 (StataCorp, College Station, Texas). Margins are statistics computed from predictions of a model at fixed values of some covariates and averaging or otherwise integrating over the remaining covariates.

### Ethical statement

Ethical approval was sought from the University of Cape Coast Institutional Review Board to conduct the study. The purpose of the study and other details were disclosed to the authorities and participants. Oral consent was sought from participants before the study started as required by the minerals commission of Ghana. Participants were not financially induced or coerced to take part in the study. It was explained to them that their participation was voluntary.

## Results

### Descriptive and bivariate analysis

From Table 1, approximately 40.0% of participants worked in the AMM subsector while 60% were from the ASM subsector. About 39.0% of goldminers indicated they had never gone for voluntary medical screening. Miners who worked in non-production departments (eg. administrators, gold buying etc) were 33.0% while 67.0% of them worked in production related departments (eg. drilling, crushing, underground, digging etc). Overall, 17.8%, 49.8% and 41.8% reported poor health, safety and environmental conditions respectively.

**Table 1:** Demographic characteristics of artisanal goldminers.

Variables	Number (%)
**Age**	
18–24 years	166 (33.2)
25–34 years	239 (47.8)
35–54 years	67 (13.4)
Above 55 years	28 (5.6)
**Gender**	
Male	432 (86.4)
Female	68 (13.6)
**Marital Status**	
Single	332 (66.4)
Married	168 (33.6)
**Education**	
No Formal Education	202 (40.4)
High school	129 (25.8)
Tertiary	169 (33.8)
**Experience**	
1–5 years	287 (57.4)
6–10 years	116 (23.2)
Above 10 years	97 (19.4)
**Monthly Income ($)**	
0–173	117 (24.8)
174–347	260 (55.2)
348–521	41 (8.7)
Above 521	53 (11.3)
**Medical Screening**	
No	195 (39.0)
Yes	305 (61.0)
**Department**	
Production	335 (67.0)
Non-production	165 (33.0)
**Sub sector**	
ASM	300 (60.0)
AMM	200 (40.0)
**Health conditions**	
Poor	89 (17.8)
Good	411 (82.2)
**Safety conditions**	
Poor	249 (49.8)
Good	251 (50.2)
**Environmental conditions**	
Poor	209 (41.8)
Good	291 (58.2)

Table 2 presents the distribution of respondents characteristics and PPE use. Notably across subsectors, a larger proportion of AMM workers indicated they use PPEs at work while only 62.7% of ASM workers use PPEs. Across departments in the subsectors, 67.2% of miners who worked in production related departments did not use PPEs, however, 98.2% of miners in the non-production related department used PPEs. Also, 95.4% of goldminers who periodically undergo voluntary medical screening indicated PPE use.

**Table 2:** Percentage distribution of PPE use by predictor variables.

Variables	PPE use		Inferential statistics	
	No (%)	Yes (%)		
**Health conditions χ^2^ (1) = 89.7, p<0.001**				
Poor	54 (60.67)	35 (39.33)	Cramér’s V = 0.4236	
Good	59 (14.36)	352 (85.64)		
**Safety conditions χ^2^ (1) = 117.7, p<0.001**				
Poor	107 (42.97)	142 (57.03)	Cramér’s V = 0.4851	
Good	6 (2.39)	245 (97.61)		
**Environmental conditions χ^2^ (1) = 121.1, p<0.001**				
Poor	98 (46.89)	111 (53.11)	Cramér’s V = 0.4922	
Good	15 (5.15)	276 (94.85)		
**Age χ^2^ (3) = 26.6, p<0.001**				
18–24 years	59 (35.54)	107 (64.46)	Cramér’s V = 0.2307	
25–34 years	42 (17.57)	197 (82.43)		
35–54 years	11 (16.42)	56 (83.58)		
Above 55 years	1 (3.57)	27 (96.43)		
**Gender χ^2^ (1) = 33.8, p<0.001**				
Male	79 (18.29)	353 (81.71)	Cramér’s V = 0.2599	
Female	34 (50)	34 (50)		
**Marital status χ^2^ (1) = 1.8, p = 0.177**				
Single	81 (24.4)	251 (75.6)	Cramér’s V = 0.0604	
Married	32 (19.05)	136 (80.95)		
**Education χ^2^ (2) = 62.7, p<0.001**				
No Formal Education	82 (40.59)	120 (59.41)	Cramér’s V = 0.3543	
Senior High	13 (10.08)	116 (89.92)		
Tertiary	18 (10.65)	151 (89.35)		
**Experience χ^2^ (2) = 38.1, p<0.001**				
1–5 years	93 (32.40)	194 (67.6)	Cramér’s V = 0.2759	
6–10 years	14 (12.07)	102 (87.93)		
Above 10 years	6 (6.19)	91 (93.81)		
**Monthly Income ($) χ^2^ (3) = 28.9, p<0.001**				
Below 174	38 (32.48)	79 (67.52)	Cramér’s V = 0.2477	
174–347	72 (27.69)	188 (72.31)		
348–521	1 (2.44)	40 (97.56)		
Above 521	2 (3.77)	51 (96.23)		
**Medical Screening χ^2^ (1) = 145.0, p<0.001**				
No	99 (50.77)	96 (49.23)	Cramér’s V = 0.5385	
Yes	14 (4.59)	291 (95.41)		
**Department χ^2^ (1) = 60.8, p<0.001**				
Production	110 (32.84)	225 (67.16)	Cramér’s V = 0.3487	
Non-production	3 (1.82)	162 (98.18)		
**Subsector χ^2^ (1) = 93.1, p<0.001**				
ASM	112 (37.33)	188 (62.67)	Cramér’s V = 0.4314	
AMM	1 (0.50)	199 (99.5)		

Table 2 also presents Pearson’s chi-square test of independence. Pearson’s chi-square and Cramer’s V statistics were used to determine whether the observed differences in occupational factors (HSE conditions) and PPE use as well as compositional and contextual factors were independent. There were statistically significant associations between health conditions (χ^2^ (1) = 89.7, p<0.001), safety conditions (χ^2^ (1) = 117.7, p<0.001), environmental conditions (χ^2^ (1) = 121.1, p<0.001) and PPE use. Cramer’s V statistic in this instance indicated moderately strong associations.

For the compositional variables, age (χ^2^ (3) = 26.6, p<0.001), gender (χ^2^ (1) = 33.8, p<0.001), education (χ^2^ (2) = 62.7, p<0.001), experience (χ^2^ (2) = 38.1, p<0.001), monthly income (χ^2^ (3) = 28.9, p<0.001) and routine medical screening (χ^2^ (1) = 145.0, p<0.001) had statistically significant association with PPE use. There was however no association between marital status (χ^2^ (1) = 1.8, p = 0.177) and PPE use. This suggests that marital status did not systematically differ with PPE use at work. Cramer’s V statistics in this instance indicated weak association for age, gender, experience, monthly income and moderately strong association for education. There was however a strong association for medical screening.

With the contextual factors, department (χ^2^ (1) = 60.8, p<0.001) and subsector (χ^2^ (1) = 93.1, p<0.001) had statistically significant association with PPE use. This meant that PPE use systematically differs across departments and subsector of artisanal goldminers in Ghana. Cramer’s V statistic indicated a moderately strong association between department, subsector and PPE use.

Based on Cramer’s V statistics, the strength of the association between the categories of each predictor and PPE use at work in increasing order of magnitude, is as follows: age< monthly income< gender< experience< department< education< health conditions< subsector< safety conditions< environmental conditions< medical screening.

### Multivariate analyses

In the multivariate analyses, three models; occupational (model 1), compositional (model 2), and contextual (model 3), were developed to assess their relationship with PPE use at work by artisanal goldminers, as shown in Table 3. In the occupational factors model, health conditions (OR = 2.11, p<0.001), safety conditions (OR = 2.22, p<0.001) and environmental conditions (OR = 2.00, p<0.05) were all statistically significant predictors of PPE use. This meant that goldminers who reported good HSE conditions were more likely to use PPEs as compared to those who reported poor HSE conditions.

**Table 3:** Complementary log-log regression model predicting PPE use by artisanal goldminers.

Variables	Model 1: Occupational factors					Model 2: Occupational factors + Compositional factors					Model 3: Occupational factors +Compositional factors +Contextual factors				
	OR	Robust SE	p-value	Conf. Interval		OR	Robust SE	p-value	Conf. Interval		OR	Robust SE	p-value	Conf. Interval	
**Health conditions (ref: Poor)**															
Good	2.11	0.408	**<0.001**	1.440	3.079	2.00	0.486	**0.004**	1.240	3.217	1.65	0.408	**0.042**	1.019	2.680
**Safety conditions (ref: Poor)**															
Good	2.22	0.443	**<0.001**	1.497	3.279	2.81	0.753	**<0.001**	1.661	4.750	1.94	0.593	**0.031**	1.062	3.528
**Environmental conditions (ref: Poor)**															
Good	1.95	0.377	**0.001**	1.337	2.850	1.02	0.225	0.939	0.660	1.569	0.87	0.209	0.555	0.542	1.390
**Age (ref: 18–24 years)**															
25–34 years						0.92	0.200	0.693	0.598	1.407	0.86	0.210	0.548	0.537	1.391
35–54 years						0.98	0.332	0.945	0.502	1.902	0.93	0.321	0.824	0.469	1.827
Above 55 years						1.39	0.760	0.548	0.475	4.061	1.53	0.799	0.413	0.552	4.259
**Gender (ref: Male)**															
Female						0.37	0.082	**<0.001**	0.243	0.574	0.16	0.064	**<0.001**	0.071	0.350
**Marital Status (ref: Single)**															
Married						1.13	0.254	0.598	0.724	1.753	1.06	0.261	0.820	0.652	1.715
**Education (ref: No Formal Education)**															
Senior High						1.97	0.433	**0.002**	1.284	3.035	1.76	0.404	**0.014**	1.121	2.759
Tertiary						2.59	0.585	**<0.001**	1.658	4.028	1.88	0.556	**0.034**	1.049	3.352
**Experience (ref: 1-5years)**															
6–10 years						1.42	0.298	0.095	0.941	2.144	1.34	0.321	0.217	0.840	2.147
Above 10 years						2.79	0.784	**<0.001**	1.606	4.836	2.73	0.792	**0.001**	1.550	4.824
**Monthly Income (ref: below $174)**															
174–347						0.41	0.101	**<0.001**	0.252	0.661	0.40	0.121	**0.003**	0.220	0.725
348–521						0.53	0.215	0.118	0.239	1.175	0.20	0.121	**0.007**	0.064	0.653
Above 521						0.55	0.198	0.098	0.273	1.116	0.29	0.140	**0.010**	0.116	0.746
**Medical Screening (ref: No)**															
Yes						2.15	0.371	**<0.001**	1.536	3.019	2.53	0.509	**<0.001**	1.708	3.755
**Department (ref: Production)**															
Non-production											2.00	0.577	**0.016**	1.137	3.521
**Subsector (ref: ASM)**															
AMM											5.31	3.282	**0.007**	1.581	17.833

When compositional factors were adjusted for in model 2, interesting trends were noticed. It was conspicuous that the compositional factors had suppressed the relationship between PPE use and environmental conditions. This clearly indicates that compositional factors completely mediate this relationship. Health conditions (OR = 2.00, p<0.05) and safety conditions (OR = 2.81, p<0.001) remained robust in predicting PPE use.

In the same compositional model, gender, education and medical screening were statistically significant in predicting PPE use. Female goldminers (OR = 0.37, p<0.001) were less likely to use PPE’s as compared to their male co-workers. Also, artisanal goldminers who had senior high (OR = 1.97, p<0.05) or tertiary education (OR = 2.59, p<0.001) were more probable to use PPE’s as compared to miners with no formal education. Likewise, miners who regularly went for medical screening (OR = 2.15, p<0.001) were more likely to use PPE’s than miners who did not go for checkups. Miners who earned between $174-$347 (OR = 0.41, p<0.001) were less likely to use PPEs as compared to those who earned less than $174 monthly. Similarly, miners who had worked for more than 10 years (OR = 2.79, p<0.001) in the mining sector were more probable than their counterparts who had just 1–5 years’ experience to use PPEs. Age and marital status had no statistically significant association with PPE use.

In model 3, where contextual factors were adjusted for, health (OR = 1.65, p<0.05) and safety (OR = 1.94, p<0.05) conditions were still robust in predicting PPE use just as observed in model 1 and 2. For the compositional factors, the relationship between gender, education, routine medical screening and PPE use remained robust and persisted. In this same model, a new relationship between monthly income and PPE use emerged, indicating mediation in the contextual model. Miners who earned $174-$347(OR = 0.40, p<0.05), $348-$521 (OR = 0.20, p<0.05) and above $521(OR = 0.29, p<0.05) were all less likely to use PPEs as compared to miners who earned less than $174. For the contextual factors, department (OR = 2.00, p<0.05) and subsector (OR = 5.31, p<0.05) were statistically significant in predicting PPE use at work. In this instance, artisanal miners who worked in non-production areas and those in AMM subsector were more likely to use PPEs as compared to their production department and ASM counterparts.

### Predictive probabilities of predictors of PPE use in artisanal mining

The margins for the predictors of PPE use in model 3 were computed to estimate predictive probabilities of responses for specified values of covariates as shown in Table 4. The results showed that the expected PPE use was 0.757 (76%) and the better the occupational conditions, the higher the probability of using PPEs. Based on the model with occupational, compositional and contextual variables on PPE use: 0.692 (69%) would be the average probability of PPE use if every miner worked under poor health conditions and 0.778 (78%) if every miner worked under good health conditions; 0.730 (73%) would be the average probability of PPE use if every miner worked under poor safety conditions and 0.835 (84%) if every miner worked under good safety conditions.

**Table 4:** Predictive probabilities of independent variables to PPE use in artisanal mining.

Variables	Margins	SE	p value	conf. Interval	
**Health Conditions**					
Poor	0.692	0.039	<0.001	0.616	0.769
Good	0.778	0.015	<0.001	0.748	0.807
**Safety Conditions**					
Poor	0.730	0.020	<0.001	0.691	0.769
Good	0.835	0.035	<0.001	0.766	0.904
**Environmental Conditions**					
Poor	0.765	0.016	<0.001	0.733	0.796
Good	0.743	0.030	<0.001	0.684	0.801
**Gender**					
Male	0.786	0.017	<0.001	0.752	0.819
Female	0.471	0.045	<0.001	0.383	0.560
**Education**					
No formal Education	0.712	0.023	<0.001	0.668	0.757
High school	0.803	0.025	<0.001	0.754	0.852
Tertiary	0.812	0.033	<0.001	0.748	0.876
**Experience**					
1–5 years	0.701	0.021	<0.001	0.660	0.742
6–10 years	0.752	0.035	<0.001	0.683	0.821
Above 10 years	0.859	0.030	<0.001	0.800	0.917
**Income ($)**					
Below 174	0.858	0.026	<0.001	0.807	0.909
174–347	0.739	0.015	<0.001	0.710	0.769
348–521	0.630	0.075	<0.001	0.484	0.777
Above 521	0.691	0.053	<0.001	0.586	0.795
**Routine medical Screening**					
No	0.673	0.028	<0.001	0.618	0.728
Yes	0.844	0.021	<0.001	0.804	0.885
**Department**					
Production	0.746	0.015	<0.001	0.717	0.776
Non-production	0.848	0.034	<0.001	0.781	0.915
**Subsector**					
ASM	0.715	0.027	<0.001	0.662	0.767
AMM	0.935	0.038	<0.001	0.860	1.009

For the compositional factors, 0.786 (79%) would be the average probability of PPE use if all miners were males and 0.471 (47%) if all miners were females; 0.712 (71%) would be the average probability of PPE use if all miners had no formal education, 0.803 (80%) if all miners had senior high education and 0.812 (81%) if all miners had tertiary education; 0.701 (70%) would be the average probability of PPE use if all miners had 1–5 years’ experience, 0.752 (75%) if all miners had 6–10 years’ experience and 0.859 (86%) if all miners had above 10 years’ experience; 0.858 (86%) would be the average probability of PPE use if all miners earned below $175 monthly and less than 0.739 (74%) if all miners earned more than $175 monthly; 0.637 (64%) would be the average probability of PPE use if all miners do not routinely go for medical screening and 0.844 (84%) if all miners go for routine medical screening.

For the contextual factors, 0.746 (75%) would be the average probability of PPE use if all miners worked in production departments and 0.848 (85%) if all miners worked in non-production departments; 0.715 (72%) would be the average probability of PPE use if all miners were in ASM subsector and 0.935 (94%) if all miners were in the AMM subsector.

## Discussion

Developing and enforcing relevant sector related HSE regulations are major factors in managing the threats in mining. However, the development and implementation of such policies have lagged behind in low-and-middle income countries such as Ghana [17]. In this instance, goldminers ability to perceive danger and act towards the perceived danger in a positive way (self-protection) is key to ensuring a healthy and safe workforce. It is widely reported that mine workers who have high compliance to HSE standards are less likely to report occupational related health challenges [16]. To understand this phenomenon, this study was carried out to examine the effects of occupational (HSE conditions), compositional and the contextual factors on PPE use among artisanal miners in Ghana.

Our findings revealed that, environmental conditions had very minimal impact (mediated by compositional and contextual factors) on artisanal goldminers use of PPEs at work. It was also established that health conditions and safety conditions were major determinants of PPE use and the better the occupational conditions the higher the probabilities of using PPEs. The significant statistical relationships between health conditions, safety conditions and PPE use were robust and persisted in all models. This meant that artisanal miners who worked in good health and safety conditions were more probable to use PPEs at work as compared to those who worked in poor health and safety conditions. It was however surprising that goldminers who worked under poor health and safety conditions were inclined to not protect themselves regularly at work. Ordinarily, it is expected that people who work in poorer conditions will instinctively protect themselves but that was not the case in this study. It is known that exposure to multiple hazards has the tendency of clouding one’s judgment of risk, usually, based on factors such as years of experience in the mining industry and the perceived potential consequences of the risk [18]. Artisanal goldminers in Ghana, who work under poor health and safety conditions, have been repeatedly exposed to hazards of their occupation to the extent that most of them no longer perceive such risks to be high. Some goldminers were visibly seen entering tunnels without helmets while others were seen in the same instance without protective boots. Some were also observed working around crushers without nose masks but rather with pieces of rags tied around the nose just below the eyes. When asked about their choices, some claimed that they had been doing it for several years without any health consequence. Others too made known the fact that there were unavailable PPEs and even when available they were inadequate.

Among the compositional factors, gender, education, monthly income, years of experience and medical screening had significant associations with PPE use. Female miners and miners who earned more monthly income were less probable to use PPEs while the highly educated, experienced and miners who routinely go for medical screening had higher probabilities of using PPEs. These findings are similar to [19–22], who posited that, compositional factors such as gender, education, monthly income and experience affect miners’ attitudes and their concepts of health and safety risks.

In the artisanal goldmining industry in Ghana, it is widely known that gender discrimination exist, especially in the ASM subsector. Male and female goldminers have unequal access to both financial and work resources. Female goldminers in this study were less probable to use PPEs as compared to their male counterparts. This finding is supported by [23] who discovered that of the 75.3% of artisanal miners who had PPEs for protection at work in the Tarkwa area, 68.7% was for men as compared to 31.3% for women. This is an indication of discrimination since the company prioritizes the health and safety of men at the expense of the women. Some female respondents in this study indicated that other female colleagues had been put in departments perceived as “not dangerous” hence, the refusal to provide adequate PPEs for them. It was also noted that because of this decision to relegate them, most women were not paid enough to acquire their own PPEs. Also, some female AMM workers who worked in offices were seen with little or no protective apparels.

Our findings also indicated that artisanal miners who had senior high and tertiary level education had higher probabilities of PPE use at work. This finding is similar to [11, 24] who reported that educated people perceived higher risk than the less educated. This is perhaps due to the fact that education increases goldminers access to information on hazards and risk at the workplace. Also in this study, artisanal miners who go for routine medical screening had higher probabilities of PPE use. Miners who routinely go for medical screening are expected to have a high self-care culture and appreciable knowledge levels of the risks involved in the mining activity, hence their desire to protect themselves at work.

Our study also found that miners who earned more income were less probable to use PPEs as compared to those who earned the least. Artisanal miners who earned $174-$347, $348-$521 and above $521 monthly, were less probable to use PPEs as compared to their counterparts who earned below $174 monthly. Ordinarily, goldminers who earn more are expected to protect themselves as compared to those who earn less. This is because high salary is a function of higher education and better choices are expected of people who are knowledgeable. In this case, workers who earned less than $174 monthly were rather concerned about their health and safety at work. It could however be argued that goldminers who earned less were less likely to afford proper health care in case of an unforeseen health outcome. This was enough reason to use available resources to ensure proper protection at work by the least paid artisanal miners.

Goldminers who had worked for more than 10 years (most experienced) had a higher probability of PPE use as compared to those who had worked for less than 5 years (least experienced). This could mean that experienced miners were more aware of and had accumulated enough knowledge of the risks associated with their work. A study in Ghana reported that old workers are usually more compliant and have positive HSE culture than younger workers [25]. Age however is a function of experience. Another study by [26] also found that younger inexperienced workers were less likely to use PPEs at work. Inexperienced workers lack the knowledge and discipline to keep themselves protected all the time albeit the poor HSE conditions some of these mine sites have. The most experienced goldminers in their long working years had witnessed colleagues, family and friends lose their lives or livelihoods from HSE accidents. Some had also experienced such accidents themselves.

On the contextual level, artisanal goldminers who worked in non-production related departments like gold buying and administrators were more probable to use PPEs as compared to those in production related areas like loading boys, equipment operators, drillers, load carriers, panners and washers. It is well documented that experienced and educated artisanal miners avoid perceived dangerous department in the sector [4, 12, 16]. This suggest that most of them are found in non-production departments. Such category of miners are known to be HSE conscious than the inexperienced, young and uneducated goldminers who prefer working in the dangerous areas where strength and brute force is required [25]. Miners with such characteristics are reckless and mostly non-compliant of HSE regulations.

Also, miners in the ASM subsector were less probable to use PPEs as compared to their AMM counterparts. The reliance on rudimentary techniques and the lack of investment in the small scale subsector exposes miners to various degrees of risks and hazards [4]. This however has the tendency to influence miners evaluation of risks and hazards. Some small scale miners in this study revealed that they do not use PPEs because they had done that for a long time without any consequences. A study by [27] also reported that concession owners and workers in the small scale subsector are profit driven and therefore do not care about the HSE conditions of their workplace or themselves. It is however not surprising that miners in the small scale subsector are less probable to protect themselves at work as compared to their counterparts in the medium scale subsector whose owners and workers think of the sustainability of their jobs.

Overall, artisanal goldmining sites with poor HSE conditions have workers who are less likely to use PPEs. The compositional and contextual characteristics of these miners mediate some of the relationships. The use of self-reported measures to assess the probabilities of PPE use in this study is a limitation. However, it is established that the concerns about self-reported measures may be exaggerated [28, 29]. It has also been well documented in literature that self-reported measures are effective for HSE studies [28–30]. The evaluation of factors that influence PPE use provide useful feedback to employees, HSE officers, managers, concession owners, NGO’s and policy makers in addressing the HSE menace in artisanal mining. In the absence of legislation, there is the need to decouple the complexities in managing HSE in artisanal mining to achieve fair and standard HSE conditions as championed by the World Health Organization. Additionally, to increase consciousness of self-protection in artisanal mining, there is the need for a national occupational health and safety policy, interventions and health promotion campaigns for better HSE conditions in artisanal mining. The findings of this study could also provide an alternative or options for the monitoring, assessment, evaluation and, application and targeting of HSE interventions in the sector.

## Conclusion

In this study, we identified important risk factors for the use of PPEs among artisanal goldminers. The findings of this study showed a conspicuous relationship between occupational (health, safety and environmental conditions), compositional (gender, education, experience, income, medical screening) contextual (department, subsector) factors and PPE use in artisanal mining. The outcome provides insights into the dynamics of the current negative HSE practices and state in the artisanal mining sector and may help in developing programs to improve the HSE culture in the sector. There are a number of adverse health implications for people who do not protect themselves in the gold mining industry, suggesting the need for a national occupational health and safety policy, interventions and health promotion campaigns, for better HSE conditions in artisanal mining. To increase safety consciousness and self-care in artisanal mining, there is the need for a national dialogue on how to improve HSE conditions and in the absence of legislation, the complexities in managing HSE in the sector need to be decoupled. Also, there is the need to pass the national occupational health and safety bill in Ghana.
